# Transient Complete Recovery of Chronic Refractory Idiopathic Thrombocytopenic Purpura after Treatment with Monoclonal Antibody Targeting SARS-CoV-2 Spike Protein

**DOI:** 10.1155/2022/8335541

**Published:** 2022-06-07

**Authors:** Pooja Gogia, Yiqing Xu

**Affiliations:** Department of Medicine, Division of Hematology/Oncology, Maimonides Medical Center, Brooklyn, NY, USA

## Abstract

Idiopathic thrombocytopenic purpura (ITP), also known as immune thrombocytopenic purpura, is an immune-mediated acquired disease characterized by transient or persistent decrease of the platelet count due to autoimmune-related destruction of platelets. Therapy for ITP relies on competing and inhibiting the autoantibody binding and destruction (intravenous immunoglobulin and anti-D immunoglobulin and spleen tyrosine kinase (Syk) inhibitor fostamatinib), augmenting platelet production (thrombopoietin receptor agonists), immunosuppression to reduce the autoantibody production, as well as splenectomy. Studies on autoantigens on the platelets suggested epitopes to be located predominantly on the GP IIb/IIIa receptor or integrin *α*IIb*β*3, though the trigger for the development of ITP is unclear. We report a case here of a 37-year-old gentleman who has chronic ITP managed on eltrombopag, who after receiving monoclonal antibody against SARS-CoV-2 (mAb) i.e. casirivimab and imdevimab for his COVID-19 infection, demonstrated complete recovery of his platelet count for a short period of time. We discuss a few potential mechanisms of action and propose further studies to elucidate the therapeutic effect of COVID-19 mAb in ITP.

## 1. Introduction

Idiopathic thrombocytopenic purpura (ITP) is an acquired autoimmune disorder characterized by transient and persistent decrease in the platelet counts (less than 100 × 10^9^/L) caused by increased platelet destruction and impaired platelet production in the absence of secondary causes [1]. The initiation of the treatment of ITP usually starts when the platelet count is below 20–30 × 10^9^/L or when the patient starts to have bleeding events. A number of treatment strategies have been developed, including glucocorticoids, immunoglobulin (IVIG), anti-D immunoglobulin, thrombopoietin receptor agonists (TPO-RAs), rituximab, spleen tyrosine kinase (Syk) inhibitor, other immunosuppressive agents, and combination therapy [2]. Splenectomy has demonstrated the greatest chance of achieving sustained remission [3].

Glucocorticoids have been reported to result in long-term remissions in only approximately 20% of cases [4]; while rituximab may be associated with a 40–60% long-term remission rate [5, 6]. The response rate of eltrombopag reaches 79%, but prolonged maintenance is required, and rebound thrombocytopenia would occur when the treatment is discontinued [7, 8]. The ITP-specific antigens have not been clearly elucidated; IVIG or anti-D immune globulin, working by interfering the Fc receptor-mediated macrophage clearance of autoantibody bound platelets, has demonstrated a response rate of 67%, which is usually temporary [9, 10].

In this report, we present a case description of a young man with a diagnosis of chronic relapsing ITP for the last 8 and half years who has been maintained on eltrombopag with improved platelet count, and showed a temporary but complete resolution of thrombocytopenia after receiving treatment with monoclonal antibodies to SARS-CoV-2 and casirivimab i.e. imdevimab, after contracting COVID-19 infection.

## 2. Case Presentation

A 37-year-old man presented in July 2021 to the emergency room (ER) with chief complaints of cough, fever, and malaise of 3 days duration and was diagnosed with SARS-CoV-2 viral infection. Eight years ago, he was first diagnosed with ITP after he had a minor nose bleed. His platelet count was 27 × 10^9^/L (normal platelet count 150–400 × 10^9^/L). He has no family history of blood disorders. His other medical history was hypothyroidism and took levothyroxine 100 micrograms a day. He denied the use of tobacco, alcohol, recreational drugs, or herbal supplements. Initial laboratory workup revealed normal coagulation parameters. ANA screen and lupus anticoagulation syndrome workup and viral infection work up including HIV antigen, hepatitis panel, *H. pylori*, hepatitis C, and Epstein-Barr IgM were all negative.

He was initially treated with pulse dose of steroids with dexamethasone 40 mg daily for 4 days and had an immediate, but short response. He was then treated with prednisone 40 mg daily with response; but his thrombocytopenia recurred with a platelet count of 18 × 10^9^/L when prednisone was tapered down to 20 mg daily. He responded to IVIG and was further treated with anti-D immunoglobulin every 2-3 months for 7 years. During that period, the peak platelet count was 122 × 10^9^/L, and the nadir was 14 × 10^9^/L. He was initiated on eltrombopag 25 mg a day, 9 months prior to the presentation in the ER. The dose was increased to 50 mg daily 3 months later, and the platelet count stabilized to 40–60 × 10^9^/L. The dose was further increased to 75 mg three months later. Fifteen days prior to the presentation, his platelet count was 62 × 10^9^/L.

On presentation to the ER, he was found to be positive by the PCR test for SARS-CoV-2 from a nasopharyngeal swab. He did not receive prior vaccination against COVID-19. He was diagnosed with COVID-19 infection of moderate severity. On physical examination, he was febrile with a temperature of 39°C, respiration rate of 18 breaths/min, heart rate 92 beats/min, and oxygen saturation 96–98% on room air. Physical exam was unremarkable except a BMI of 42. He was treated with monoclonal antibody (mAb) against SARS-CoV-2, i.e., casirivimab 600 mg and imdevimab 600 mg.

Laboratory studies on the day of ER visit prior to the mAb treatment showed platelet count of 82 × 10^9^/L and lymphopenia of 1.1 × 10^9^/L. Patient's symptoms subsided over the next 1-2 weeks. On his subsequent outpatient follow-up, 14 days after the mAb infusion, his platelet count was 292 × 10^9^/L, so eltrombopag was held. On the 17^th^ day after mAb infusion, the platelet count was 199 × 10^9^/L. On the 24^th^ day after mAb treatment, his platelet count declined to 45 × 10^9^/L, and eltrombopag was reinitiated at 50 mg daily. On the 74^th^ day after ER visit, his platelet count was 34 × 10^9^/L, and his dose of eltrombopag was increased to 75 mg daily. On the 90th day after mAb treatment, his platelet count was 73 × 10^9^/L.

**Figure 1:**
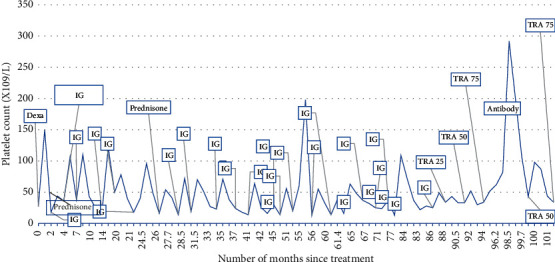
Depicting trend in platelet counts with various treatments dexa: dexamethsone (pulse dose of steroids). Prednisone: prednisone 1 mg/kg. IG: anti-D immunoglobulin. TRA (thrombopoietin receptor agonist) 25: eltrombopag 25 mg. TRA 50: eltrombopag 50 mg. TRA 75: eltrombopag 75 mg antibody: monoclonal antibody (mAb) against SARS-COV2.

The trend of his platelet counts in relationship to his treatments is shown in Figure 1.

## 3. Discussion

ITP has long been considered an autoimmune disease and autoantibody-associated platelet destruction is the mechanism of thrombocytopenia [11]. It may be associated with viral infections and a number of chronic disorders such as systemic lupus erythematosus and chronic lymphocytic leukemia [11]. Studies on uncovering the epitopes on the platelets for autoantibody production has revealed GP IIb/IIIa receptor or integrin *α*IIb*β*3 to be the predominant target. Other potential sources are GP Ib/IX, IV, and Ia/IIa; however, GP IV or Ia/IIa are never the sole targets [12, 13]. Regardless, a reliable gold standard test has not been established, and the diagnosis of ITP remains a diagnosis of exclusion [14].

The therapeutic application and success of IVIG or anti-D immune globulin corroborates the autoimmune mechanism of thrombocytopenia in ITP. IVIG interferes with the uptake of autoantibody-coated platelets by the macrophages; while anti-D immunoglobulin aims to saturate the macrophage Fc receptors with anti-D-coated red blood cells (RBCs) and therefore works to inhibit the destruction and clearance of the platelets [10]. Although a rapid therapeutic response is usually expected, its efficacy is inevitably short acting, averaging about 3-4 weeks [10].

Casirivimab and imdevimab are monoclonal antibodies that bind to different sites on the receptor-binding domain of the spike protein of SARS-CoV-2 and, thereby, block its attachment to the human ACE2 receptor and subsequent entry to the human cells [15]. The combination use has been shown to effectively decrease the progression of COVID-19 infection and to decrease the rate of hospitalization in high-risk patients [16]. It has been approved by US Food and Drug Agency (FDA) for Emergency Use Authorization to patients who have mild COVID-19 infection who do not exhibit hypoxia and who do not need hospitalization and, subsequently, also for postexposure prophylaxis for high-risk patients [16].

The observation of the improvement of platelet count in this ITP patient after contracting COVID-19 infection and receiving casirivimab and imdevimab treatment is both intriguing and fascinating. The patient had a platelet count of 80 × 10^9^/L prior to mAb treatment, and this level of platelet count was the expected response to eltrombopag. However, with a complete recovery of count to 292 × 10^9^/L after the COVID-19 monoclonal antibody treatment, it is compelling to hypothesize that the improvement in the platelet count was the result of the treatment effect from casirivimab and imdevimab, instead of the effect of eltrombopag.

Several mechanisms of action may be proposed. First, mAb might have exerted an effect with a similar mechanism as IVIG or anti-D immunoglobulin, as nonspecific competition with platelets coated with autoantibodies for the Fc receptors for macrophage-mediated destruction. We noted that the dose of immunoglobulin in the monoclonal antibody treatment (1200 mg total) was significantly lower than that in the therapeutic IVIG dose (1000 mg/kg x 2 days), and this low dose may not function effectively as a nonspecific competition mechanism.

A second possibility is specific binding and competition of the platelet-specific autoantigen by the therapeutic antibodies. In that speculation, we hypothesize that there might be shared epitopes between the autoantigens in ITP and the SARS-CoV-2 spike protein. In fact, a molecular mimicry between the spike or other proteins from the virus and the platelet specific antigens has been proposed as a mechanism of increased platelet destruction leading to thrombocytopenia in COVID-19 infection [17]. One would ask why would therapeutic antibody casirivimab and imdevimab, similarly binding to shared platelet antigens, lead to platelet stabilization, while binding from the self-produced antibody to the viral spike protein lead to platelet destruction? One may raise a hypothesis that the unique feature of the Fc fragment of those monoclonal antibodies may fail to elicit strong Fc receptor binding and clearance, different from the polyclonal Fc fragments from self-produced antibodies. In that regard, it is known that depending on the properties in the Fc region of the IgG molecule, interaction of IgG with Fc receptor can be activating or inhibitory; and a large body of preclinical studies have investigated possible alteration of effector function by modulating the amino acid sequence and glycosylation pattern in IgG Fc [18].

Third, viral particles or viral fragments coated with casirivimab or imdevimab could compete with autoantibodies for Fc receptors, thus decreasing platelet destruction.

Presumably, a simple laboratory test can be done to test the above hypothesis. Serum from patients with ITP may be added to the viral spike proteins-coated microtiter plates to test for binding. The caveats for this experiment could be low titer or low affinity of the autoantibodies to the spike protein. If this proof-of-concept experiment shows supportive result, further studies should be performed in ITP patients to test the therapeutic effect in rapid raise in platelet count with casirivimab and imdevimab.

The association of COVID-19 infection and thrombocytopenia has been well studied and is typically mild [19, 20]. Severe thrombocytopenia can be seen in COVID-19-infected patients who have preexisting ITP [21]. Multiple mechanisms of thrombocytopenia associated with COVID-19 infection have been postulated, in addition to the above-discussed molecular mimicry theory. Decreased production by infected bone marrow cells resulting in abnormal hematopoiesis and consumption by damaged lung tissues and pulmonary endothelial cells is one of them [17]. Particularly, as most of the patients show increase in the D-dimer level in the initial phase of COVID-19 infection, a consumptive process may be a more important mechanism, especially in the early stage of the COVID-19 infection, when autoantibodies have not been fully produced.

On the other hand, the vaccination with ChAdOx1 nCoV-19 vaccine may rarely elicit a different type of antibody, which targets platelet factor 4 (PF4) and causes the development of immune thrombotic thrombocytopenia, which results in thrombosis and clinically mimics autoimmune heparin-induced thrombocytopenia and thrombosis (HITT) [22, 23]. Our case scenario does not suggest an autoimmune process with that mechanism.

Eltrombopag is a thrombopoietin receptor agonist that acts on the axis of increasing the production of platelets [24]. The efficacy appears to be dose dependent, and few cases of patients going into remission without the need for unremitting treatment have been reported [7]. In a retrospective analysis of 260 patients, 201 achieved complete remission, and out of which in 62 patients, eltrombopag was discontinued without need of continuous treatment [7]. In this case, the patient responded to the increased dose of eltrombopag well with higher platelet count, and his response was as predicted. Nonetheless, he required resumption of eltrombopag treatment about 3-4 weeks after mAb treatment, which was consistent with the wearing-off of the therapeutic effect from the monoclonal antibody, rather than the remission-induction effect from eltrombopag. In addition, the time course of his decline of platelet to his baseline of 45 × 10^9^/L on day 24 from mAb treatment is consistent with the time course of the effect seen after IVIG treatment.

In conclusion, we report an interesting case of the possible therapeutic effect on ITP from monoclonal antibody developed for COVID-19 treatment, i.e., casirivimab and imdevimab. This report may be the first case report in this aspect. The impressive result prompted us to hypothesize various possible mechanisms, including nonspecific competition of mAbs with platelet-specific autoantibodies for Fc receptor binding and macrophage clearance; specific competitive binding of molecularly similar platelet autoantigen leading to platelet stabilization and competition of viral particles coated with therapeutic mAb with autoantibodies for Fc receptors-mediated clearance. This notion requires further careful preclinical and clinical studies, and a prospective study to examine the therapeutic effects of mAb in thrombocytopenia associated ITP may be considered.
